# Application of the targeted sequencing approach reveals the single nucleotide polymorphism (SNP) repertoire in microRNA genes in the pig genome

**DOI:** 10.1038/s41598-021-89363-5

**Published:** 2021-05-10

**Authors:** Klaudia Pawlina-Tyszko, Ewelina Semik-Gurgul, Artur Gurgul, Maria Oczkowicz, Tomasz Szmatoła, Monika Bugno-Poniewierska

**Affiliations:** 1 Department of Animal Molecular Biology, National Research Institute of Animal Production, Krakowska 1, Balice, 32-083 Kraków, Poland; 2 Center for Experimental and Innovative Medicine, The University of Agriculture in Kraków, Rędzina 1c, 30-248, Kraków, Poland; 3 Department of Animal Reproduction, Anatomy and Genomics, The University of Agriculture in Kraków, al. Mickiewicza 24/28, 30-059 Kraków, Poland

**Keywords:** Agricultural genetics, miRNAs, Genetics, Genomics

## Abstract

MicroRNAs (miRNAs) are recognized as gene expression regulators, indirectly orchestrating a plethora of biological processes. Single nucleotide polymorphism (SNP), one of the most common genetic variations in the genome, is established to affect miRNA functioning and influence complex traits and diseases. SNPs in miRNAs have also been associated with important production traits in livestock. Thus, the aim of our study was to reveal the SNP variability of miRNA genes in the genome of the pig, which is a significant farm animal and large-mammal human model. To this end, we applied the targeted sequencing approach, enabling deep sequencing of specified genomic regions. As a result, 73 SNPs localized in 50 distinct pre-miRNAs were identified. In silico analysis revealed that many of the identified SNPs influenced the structure and energy of the hairpin precursors. Moreover, SNPs localized in the seed regions were shown to alter targeted genes and, as a result, enrich different biological pathways. The obtained results corroborate a significant impact of SNPs on the miRNA processing and broaden the state of knowledge in the field of animal genomics. We also report the targeted sequencing approach to be a promising alternative for the whole genome sequencing in miRNA genes focused studies.

## Introduction

MicroRNAs are classified as short, non-coding RNAs, which do not code for proteins but instead orchestrate gene expression at the post-transcriptional level. They are transcribed as precursor sequences, and their maturation involves two significant steps. A long primary miRNA transcript (pri-miRNA), transcribed from a miRNA gene, is cleaved by the Drosha/DGCR8 enzyme complex to give rise to the hairpin precursor (pre-miRNA)^1^. A pre-miRNA undergoes a second cleavage by the Dicer enzyme, which results in miRNA duplex release^2^. One strand of this duplex, termed the guide, is incorporated into effector RNA-induced Silencing Complex (RISC) along with Argonaute protein^3,4^. RISC exerts its influence on gene transcripts via sequence complementarity of a loaded miRNA and 3′UTR of an mRNA. It may result in its translational repression and less frequently mRNA degradation^5^, or in some cases, the upregulation of expression^6^, influencing the further synthesis of a protein. On the one hand, one microRNA may have multiple target genes, and on the other hand, one transcript can be regulated by different miRNAs. Because of such an extensive and multifaceted network of regulation, miRNAs play vital roles in myriads of biological processes and molecular functions, and, as a result, are frequently reported to be deregulated in a variety of diseases, such as, e.g. stroke, cancer, or major depression^5,7–10^.

Single nucleotide polymorphism (SNP), one of the most common genetic variations in the genome^11^, SNPs have been shown to profoundly influence a gene function and regulation^12^. MiRNA genes are no exception, and numerous studies have revealed that SNPs substantially affect microRNA processing, stability, and functioning^13–16^. It is effectuated in two ways, namely via SNP polymorphisms within the sequences of target genes, and miRNA genes. SNPs occurring in a target mRNA may cause target alterations since they may lead to the gain or loss of a miRNA binding site or the creation of a binding site for a new miRNA^13,17^. Similarly, SNPs located in pre-miRNAs may create a new binding site, cause the loss of existing ones as well as create a binding site for new target genes.

Thus, the occurrence of an SNP may substantially change the interaction profile of a miRNA with its target genes, and, as a result, influence the whole downstream biological pathways that can be related with, e.g. intra-individual variation and disease susceptibility. It has been evidenced by a handful of reports showing the influence of SNPs in miRNAs and their targets on complex traits and diseases^18–25^. For example, an SNP located in pre-miR-146a (rs2910164) was established to be associated with the expression downregulation of this miRNA^20,21^. Furthermore, findings are confirming the association of an SNP present in miR-499 (rs3746444) with various types of cancer^22,24,25^.

In livestock, SNPs in miRNAs and their target genes are also found to be associated with significant production traits, such as growth, fatness, and meat traits^26–29^, however, the number of such studies is very scarce. It may stem from i.a. a limited number of identified miRNA SNPs. For example, 1552 polymorphic human pre-miRNA regions were deposited in the miRNA SNiPer v. 4.0 database, whereas this number amounted to 741 and 89 in cattle and pigs, respectively^30^. The domestic pig is of the utmost importance in the meat production, and, what is more, it constitutes an excellent, alternate, large mammal model organism for the human because of the similarity of the development, physiology, and anatomy^31–33^. Hence, a further investigation of the variability of miRNA genes in this species not only will contribute to the development of biomarkers associated with traits variability, but also may serve as source information for comparative studies.

To accelerate progress in this area, analyses on the genome-scale are necessary. It can be achieved by implementing high-throughput technologies such as next-generation sequencing (NGS). This method has a broad range of applications and advantages; however, it may generate substantial costs in the case of whole-genome sequencing. Therefore, we attempted to apply the targeted sequencing approach, which enables deep sequencing of chosen genomic regions of interest, to reveal SNP variability of miRNA genes in the pig genome for the first time. To this end, we used NimbleGen SeqCap EZ Enrichment System (Roche), which utilizes a tiling approach for probe design and is characterized by high enrichment efficiency. The obtained results not only provide information on the usefulness of this technology in similar studies but also allow better genomic characterization of the pig in terms of SNP-miRNA variability, and provide potential markers useful for association studies with crucial production traits. Moreover, such a comprehensive analysis of SNP characteristics of miRNA genes from the global, genomic point of view will constitute a basis for further interspecies research and potential applications in human diseases.

## Results

### Characteristics of the called variants

**Figure 1:**
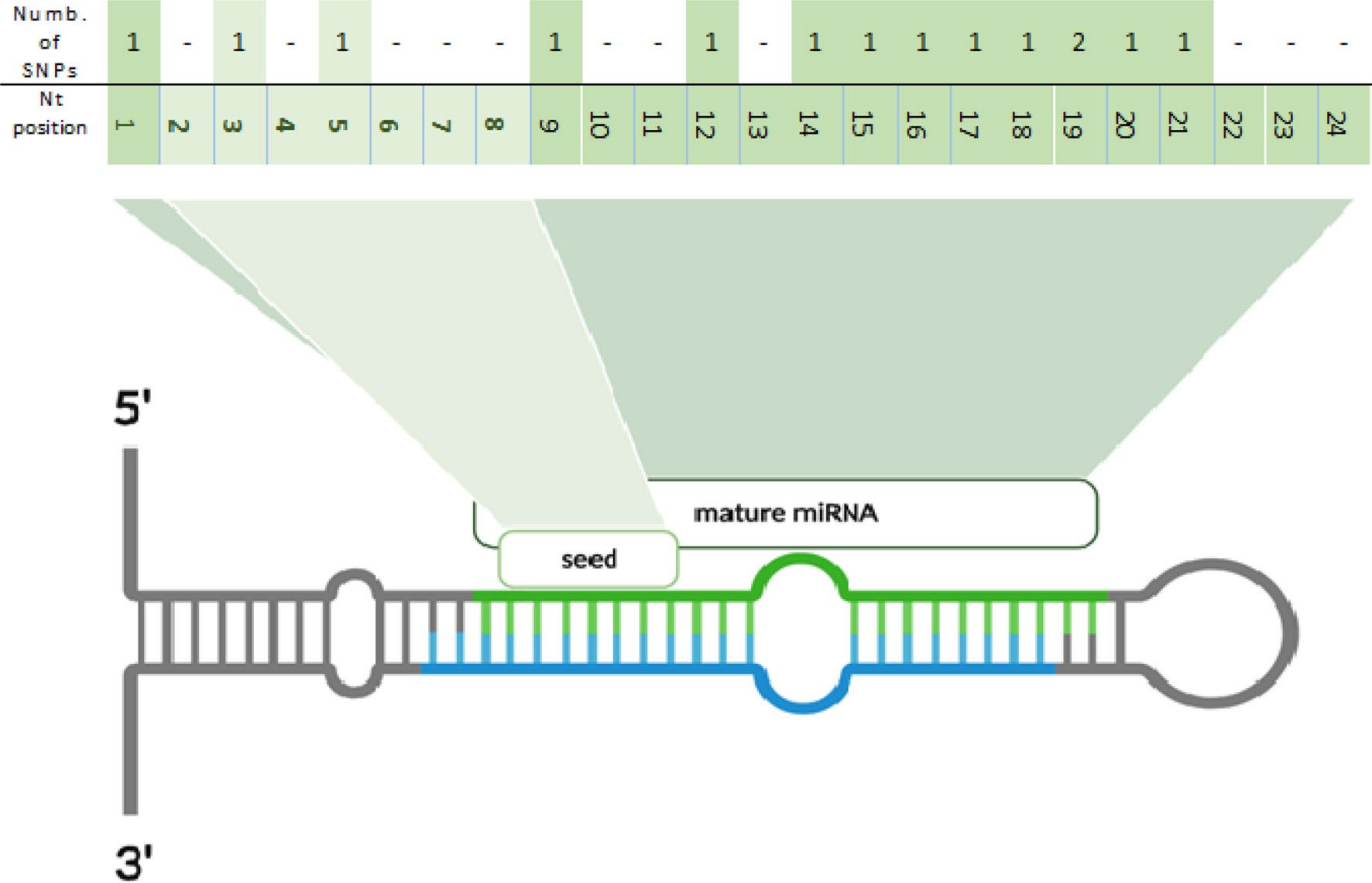
Distribution of the localization of all SNPs identified in mature miRNA sequences in this study. The upper row of the table contains data on the number of SNPs identified in a given position of the mature miRNA sequence, while the lower row indicates a nucleotide position of the mature miRNA sequence. The light green coloured cells stand for the positions of the seed nucleotides (2–8).

**Figure 2:**
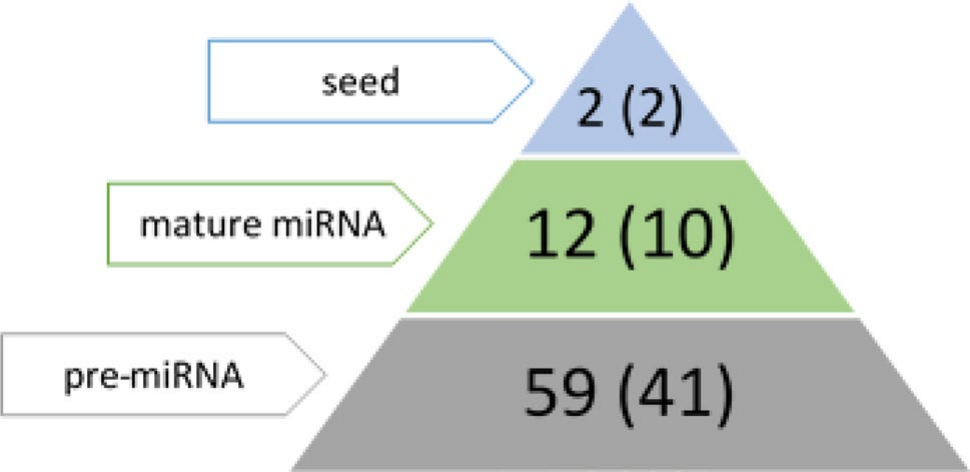
Data on the number of SNPs identified in the seed sequence (blue triangle), in the mature miRNA sequence beyond the seed (green traingle), and in the pre-miRNA sequence beyond the mature miRNA (grey triangle). Numbers in brackets denote the number of miRNAs with such SNPs.

**Figure 3:**
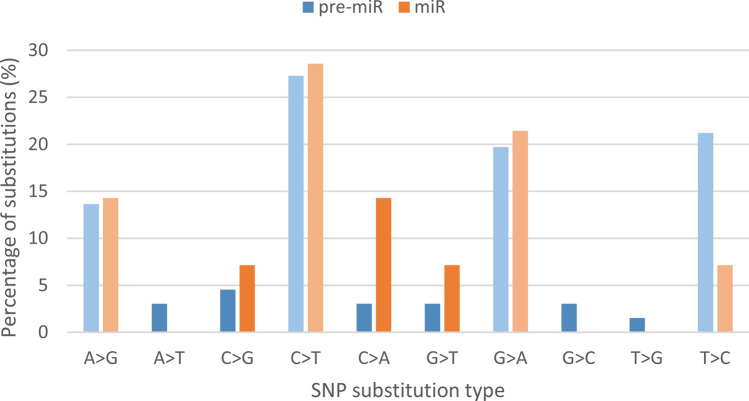
Details on the frequency of SNP substitution types identified in the whole pre-miRNA sequences (pre-miR) as well as mature miRNA sequences (miR). Lighter-coloured bars denote transitions, while darker-coloured bars stand for transversions.

The performed targeted sequencing allowed to obtain on average 7,873,079 high-quality reads per sample, of which 6,967,606 (88%) constituted on-target reads. The average depth of coverage was 57.07. The subsequent analyses revealed as many as 5,195 potential variants dispersed in the pig genome, including SNPs, deletions, insertions, and complex variants. They were identified on all pig chromosomes, and their number ranged from 95 on chromosome 8–743 on chromosome 15, including 13 variants assigned to unmapped contigs. Of the called variants, 81 were localized in pre-miRNA sequences (Supplementary File 2), including 73 SNPs. These SNPs were localized in 50 unique pre-miRNAs, which constitutes 13% of all investigated microRNA sequences. Two SNPs were localized in the miRNA seed sequence (2.7%), 12 in the mature miRNA sequence beyond the seed (16.5%) (Fig. 1), while 59 in the pre-miRNA sequence beyond the mature miRNA (80.8%) (Fig. 2). 43 pre-miRNA variants were previously identified and had database (Ensembl https://www.ensembl.org/index.html) accession numbers (Supplementary File 2). The analysis of SNP types revealed that the most common in pre-miRNA and mature miRNA sequences were transitions, specifically, C>T followed by G>A (Fig. 3). Among the transversions, C>G was the most frequent in pre-miRNAs, while C>A in miRNAs. All types of nucleotide substitutions were observed in pre-miRNA sequences, whereas no A>T, G>C and T>G substitutions were detected in mature microRNA sequences (Fig. 3).

The mean MAF for all identified SNPs amounted to 0.275 (± 0.161), while for the pre-miRNA-located SNPs was slightly lower (0.262 (± 0.165). The observed heterozygosity was equal to 0.46 on average, across all the sequenced regions as well as across only pre-miRNA regions. All detected SNPs were physically distanced from each other by 636.7 (± 2044) kb on average, while the minimal and maximal distances ranging from 1 bp to 37.7 Mb. When it comes to the physical distance of pre-miRNA-located SNPs, it was substantially higher and amounted to 19.1 (± 43.7) Mb, whereas the minimal and maximal distances oscillated between 1 bp and 280.8 Mb. The density of SNPs per 1 kb of the whole targeted region was 41, while per 1 kb of pre-miRNA sequences was 2.4.

### SNP influence on miRNA structural and functional parameters

**Figure 4:**
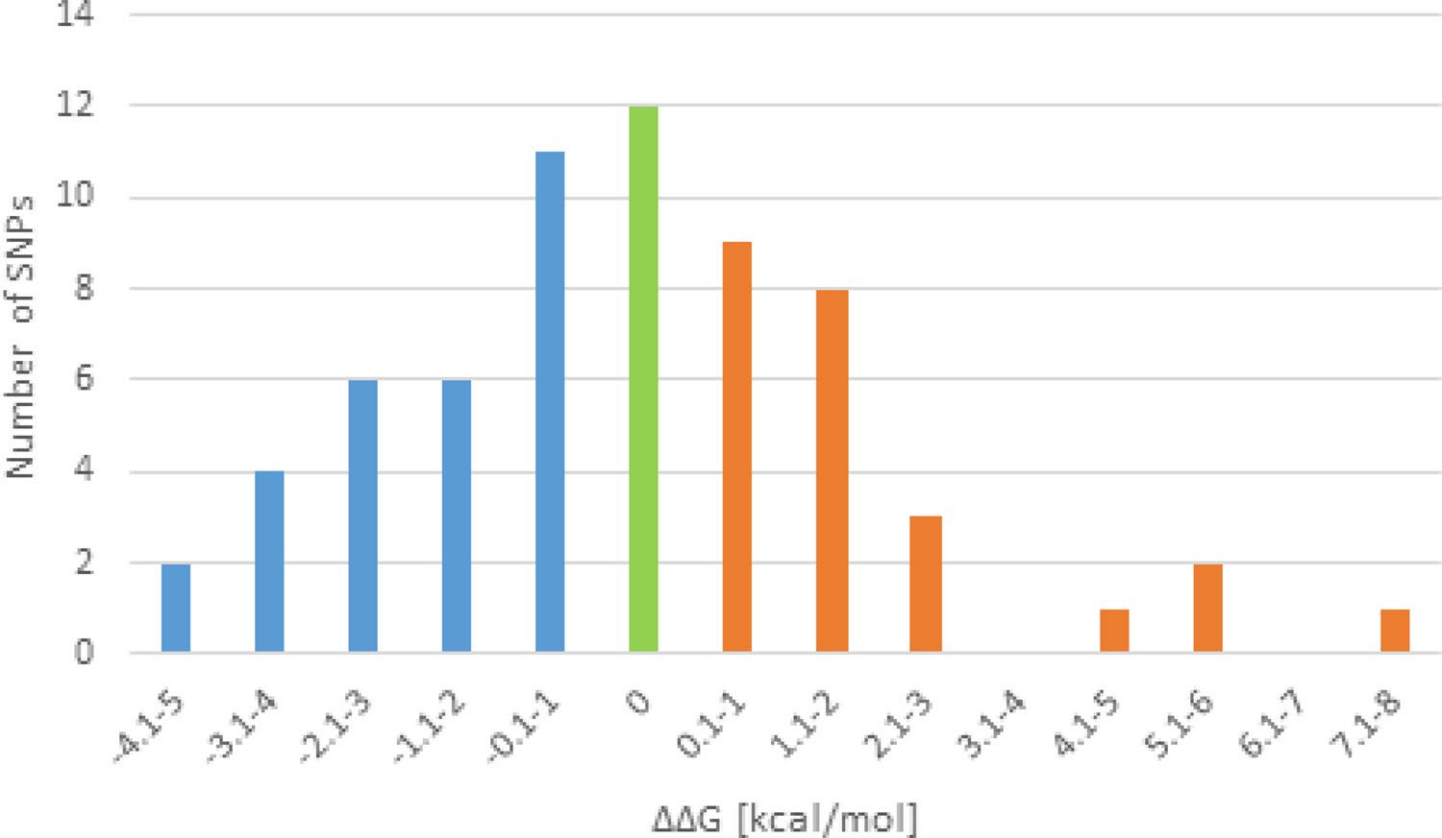
Distribution of minimal free energy changes (MFE; ΔΔG, kcal/mol) of pre-miRNA hairpin secondary structures induced by single SNPs. The blue bars denote the decrease of MFE, while the orange bars denote the increase of MFE.

To explore the impact of the SNPs on the hairpin secondary structure and its thermodynamical stability, we employed RNAfold software. One pre-miRNA was folded twice—the first time as a reference sequence and the second time after introducing an SNP. 53 SNPs elicited a non-zero minimal free energy (MFE) change (|ΔΔG|) ranging from 0.1 to 7.4 kcal/mol (Fig. 4). 29 SNPs decreased the minimal free energy of the hairpin structures, while 24 SNPs increased it (Fig. 4).

**Figure 5:**
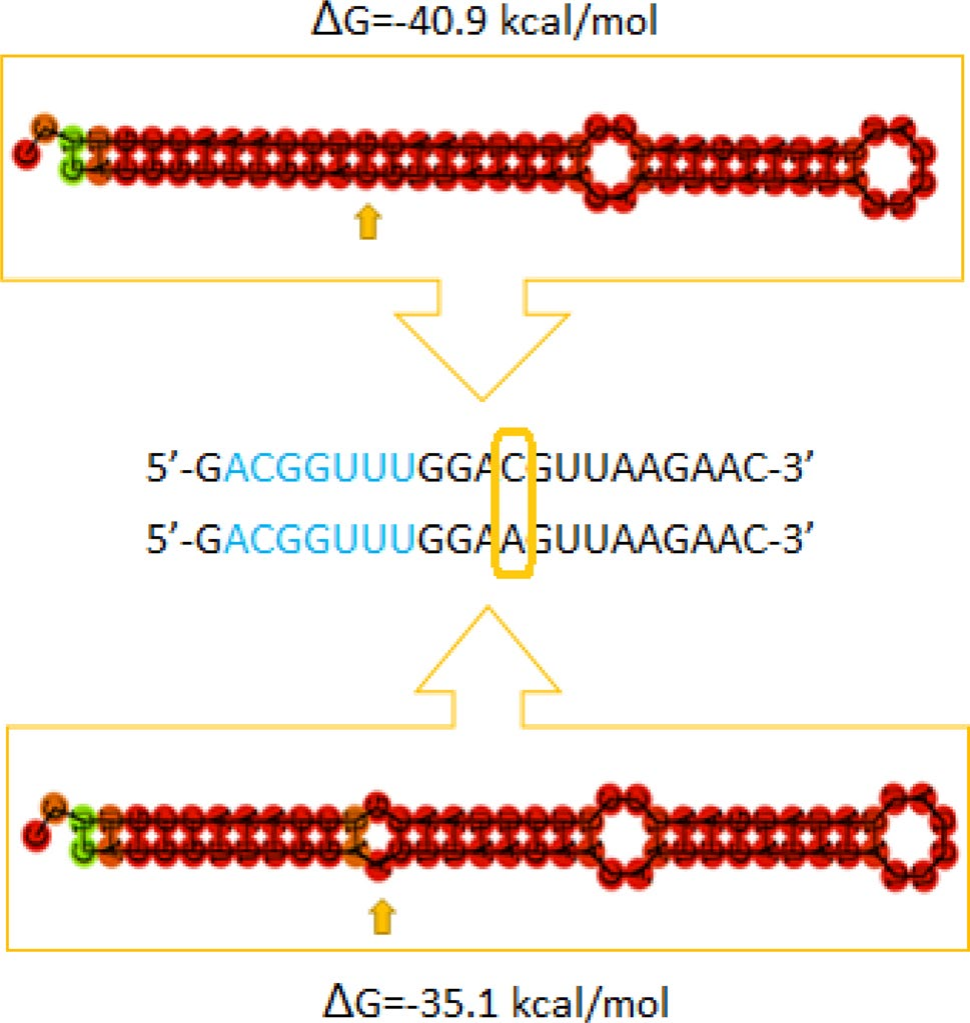
Graphical visualization of an SNP (C>A; rs334271387) identified in the sequence of ssc-miR-7141-5p and its impact on the hairpin secondary structure and its minimal free energy (MFE; ΔG). The seed region is highlighted in blue, while the SNP is framed. The yellow arrow shows the SNP localization and a bulge which it created.

**Figure 6:**
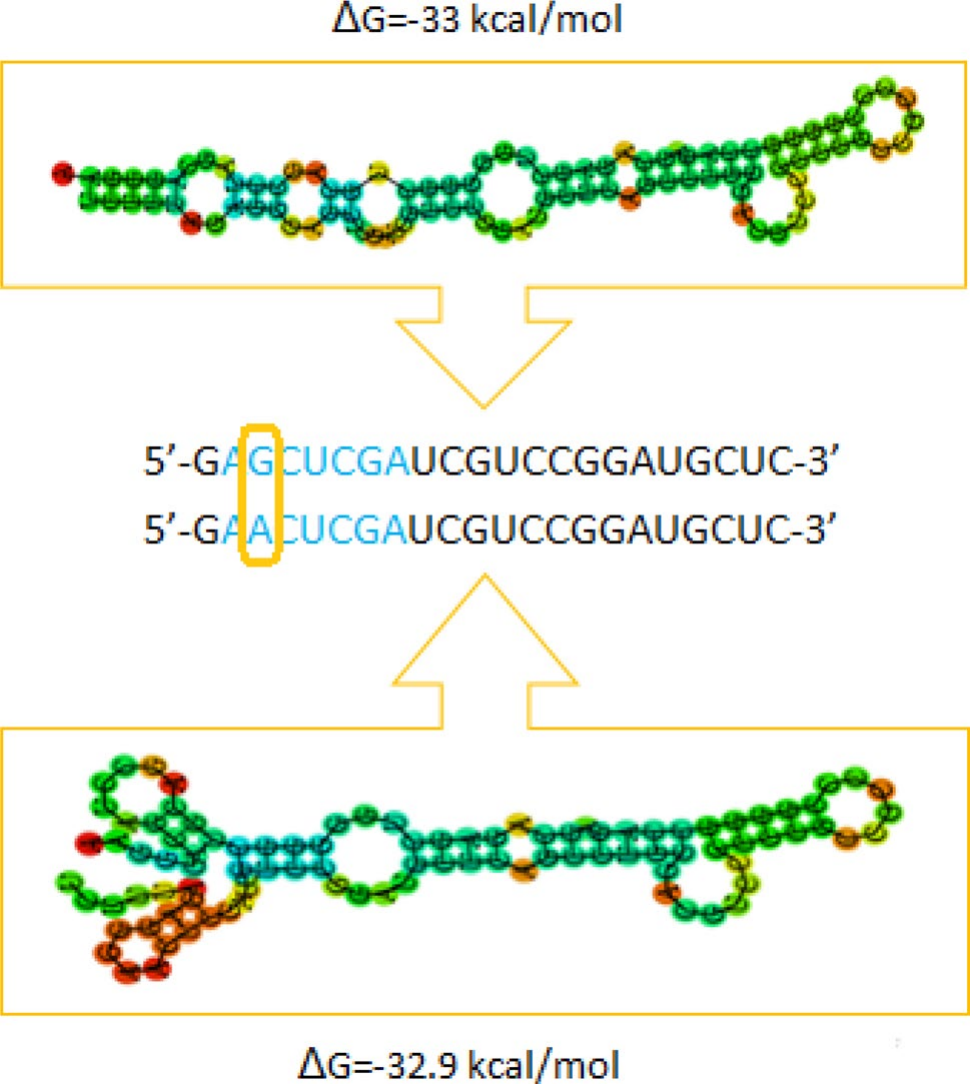
Graphical visualization of an SNP (G>A) identified in the seed region of ssc-miR-9819-5p and its impact on the hairpin secondary structure and its minimal free energy (MFE; ΔG). The seed region is highlighted in blue, while the SNP is framed.

The average energy change of a secondary structure was 1.51 kcal/mol. Moreover, 30 identified SNPs affected the hairpin structure of pre-miRNAs, with or without a change of their MFE energy. Examples are presented in Figs. 5 and 6.

**Figure 7:**
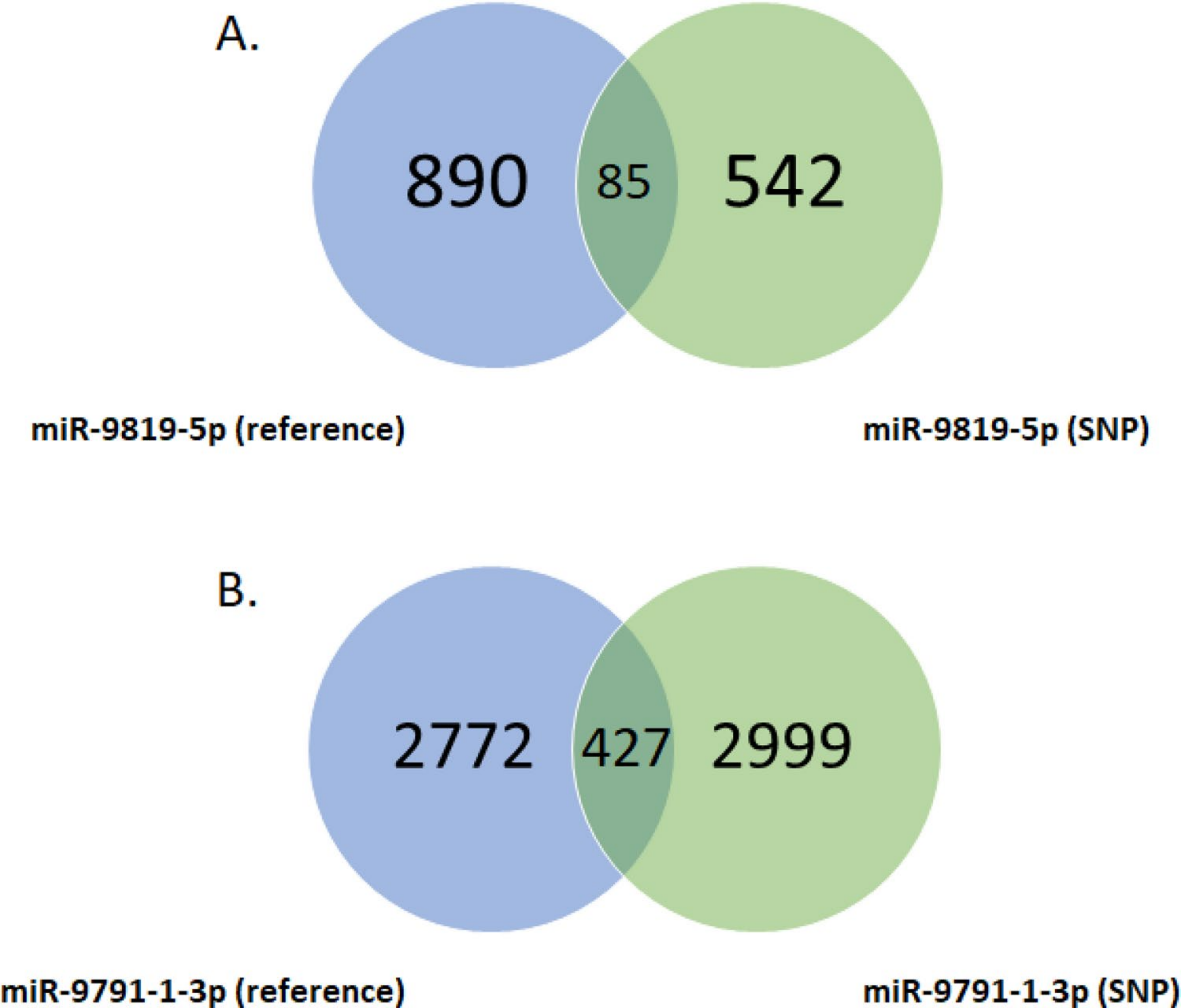
Venn diagram of target genes predicted for wild type and SNP-altered miRNA seeds: (**A**) data on the number of predicted target genes of miR-9819-5p for the reference seed (the blue circle), and the SNP-altered seed (the green circle); (**B**) data on the number of predicted target genes of miR-9791-1-3p for the reference seed (the blue circle), and the SNP-altered seed (the green circle).

**Figure 8:**
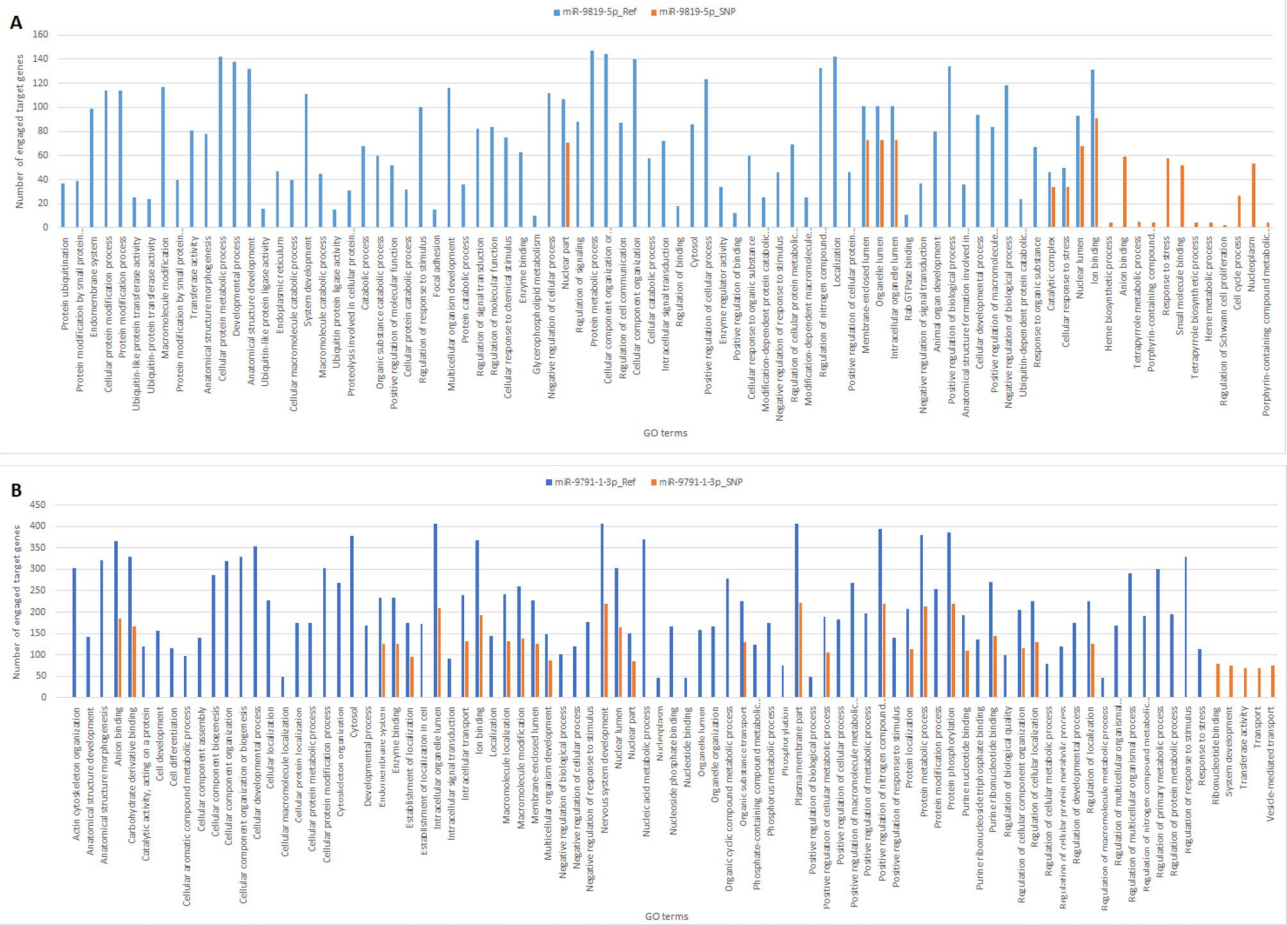
(**A**) statistically significant (p value ≤ 0.05) GO terms enriched in target genes of miR-9819-5p reference sequence (the blue bars) and miR-9819-5p with the SNP (the orange bars); (**B**) statistically significant (p value ≤ 0.06) GO terms enriched in target genes of miR-9791-1-3p reference sequence (the blue bars) and miR-9791-1-3p with the SNP (the orange bars).

Next, we performed the identification of target gene sets for two miRNAs with SNPs located in their seed regions (miR-9819-5p and miR-9791-1-3p) to explore their influence on miRNA-mRNA interaction and target gene selection. The prediction was carried out for reference seeds and seeds altered with the detected SNPs. The analysis revealed a decrease in the number of potential targets from 890 to 542 for miR-9819-5p G>A and an increase from 2772 to 2999 for miR-9791-1-3p G>A (Fig. 7). To investigate the impact of these SNPs on biological pathways via the change of target gene sets which they caused, in the next step, we performed gene ontology enrichment analysis for each target gene set (reference and SNP-altered) for both miRNAs. The analysis revealed the statistically significant (p-value ≤ 0.05) enrichment of different GO term sets for miR-9819-5p reference and SNP-altered seed (Fig. 8A) as well as for miR-9791-1-3p reference and SNP-altered sequence (Fig. 8B). Those GO terms which were common for reference and SNP-altered miRNA seeds had a lower number of engaged target genes in the case of SNP-altered seed predictions (Fig. 8).

### Results of Sanger sequencing validation

**Figure 9:**
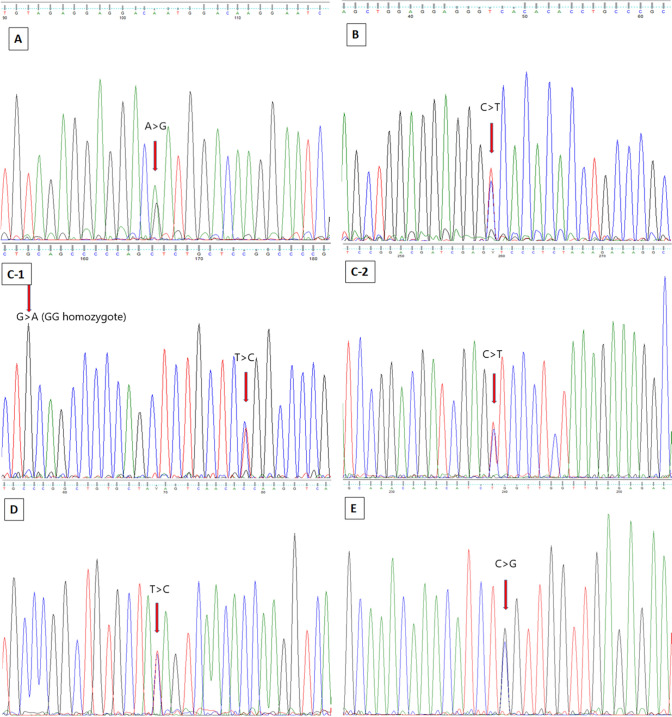
Exemplary Sanger sequencing chromatograms: (**A**) region 3, ssc-miR-9831, chr1 4108477 A>G; (**B**) region 6, ssc-miR-9853-1, chr12 57502421 C>T; (**C**) region 8, ssc-miR-9819, C-1: chr5 8348872 G>A (GG homozygote), 8348891 T>C; C-2: chr5 8348943, C>T; (**D**) region 17, ssc-miR-146b, chr 14 123301846 T>C; (**E**) region 20, ssc-miR-2483, chr X 117608224 C>G. The red arrow points to the given SNP.

We used Sanger sequencing to verify the results obtained by targeted sequencing. The 20 validated regions embraced 33 SNPs. The verification revealed a high concordance between these two methods. 18 of the validated regions, including 28 SNPs, were confirmed in heterozygous and homozygous samples (90%) (Supplementary file 3). Exemplary chromatograms are depicted in Fig. 9.

## Discussion

In this study, we characterized the SNP repertoire in miRNA genes in the pig genome with the use of the cutting-edge technology such as targeted sequencing and performed an in silico analysis of the influence of identified SNPs on miRNA secondary structure stability and target gene switching. The applied approach resulted in high accuracy, which manifested as 88% of on-target reads and 90% concordance with the alternative method results (Sanger sequencing). It is worth noting that the pig genome build was updated, and a few chromosome regions were removed while many were moved to other locations after the targeted sequencing design had been completed. This change may be one of the causes underlying 10% discrepancies of the validation results, which may also arise from the continued presence of problematic, unassigned regions. Moreover, some discrepancies between the two methods’ results may stem from different principles behind the used methods (targeted sequencing based on hybridization of DNA and Sanger sequencing based on PCR) and the fact that each technique holds its own level of false-positive and false-negative results.

As a result of the downstream analysis, 81 variants were mapped to porcine pre-miRNA sequences, of which 73 were SNPs. The analysis of their distribution showed that the lowest number of SNPs (2.7%) was localized in the miRNA seed region, responsible for target gene binding. 12 (16.5%) SNPs were called in mature miRNA sequences, beyond the seed region, while as many as 59 (80.8%) SNPs were mapped to pre-miRNA sequences beyond the mature sequence. This increasing accumulation of SNPs along with the distance from the seed region and mature sequences is in concordance with other studies and is believed to constitute the manifestation of the negative selection, because of the functional importance of the seed region in target gene recognition^30,34,35^.

We also looked closer into the identified SNPs, and analyzed the types of substitutions. We found that transitions were undeniably more common than transversions, both in pre-miRNA sequences (82% vs 18%), and mature microRNAs (71% vs 29%) (Fig. 3). It corroborates the results published by Omariba and colleagues in the mouse genome^35^. In general, the proportion of single transitions in pre-miRNA and mature miRNA sequences was similar. On the other hand, mature miRNAs showed only C>G, C>A, and G>T transversions, while also other types of transversions were observed in pre-miRNAs, which may partially stem from the higher overall number of SNPs located in pre-miRNA sequences than in mature miRNAs.

Next, we performed an in silico analysis to investigate the potential influence of the seed-located SNPs on miRNA-mRNA interactions. The analysis revealed that the introduction of the SNPs in the seed region of ssc-miR-9819-5p and ssc-miR-9791-1-3p caused a substantial change in the set of target genes. 85 target genes remained common for the reference and SNP-altered ssc-miR-9819-5p, while 805 were lost and 457 were gained (Fig. 7A). We observed a similar tendency for ssc-miR-9791–1-3p that is a loss of 2345 target genes and a gain of 2572, whereas 427 genes were targeted by both variants of this miRNA (Fig. 7B). This profound influence of SNPs on target gene switching concurs with other studies, for example, on the human^34^ and mouse^35^ genomes. Furthermore, an SNP-caused target gene switching of one microRNA, namely ssc-miR-378, was also computationally and experimentally established in the pig^28^.

To elucidate if such target gene alterations could have further consequences that is impact biological pathways and, as a result, cell functioning, we carried out Gene Ontology enrichment analysis. It showed that the alterations of target gene sets found their reflection in biological pathways since different numbers and sets of GO terms were identified for both SNP-altered seeds in comparison to the reference miRNAs (Fig. 8). Only a few GO terms were common for the reference sequences and SNP-altered sequences; however, the number of engaged target genes in the same GO term was lower in the case of the SNP-altered seeds in comparison to the reference seeds. Furthermore, both SNP-altered seeds of ssc-miR-9819-5p and ssc-miR-9791-1-3p indirectly enriched a lower number of GO terms than the reference miRNAs. It may be a consequence of the decreased number of genes targeted by the SNP-altered seeds as well as targeting genes which do not enrich biological processes at the statistically significant levels.

Among the most significant GO terms affected by the SNP introduction in the seed region of ssc-miR-9819-5p were vital cellular processes associated with i.a. protein turnover and modification (e.g. protein ubiquitination, protein modification by small protein conjugation, protein modification process, and ubiquitin-protein transferase activity), and cell metabolism (e.g. cellular protein metabolic process, cellular macromolecule catabolic process, catabolic process, glycerophospholipid metabolism). When it comes to ssc-miR-9791-1-3p, the most significant SNP-affected terms were related to i.a. cellular components organization and localization (e.g. cellular component organization or biogenesis, cellular localization, establishment of localization, organelle lumen, organelle organization), and key cellular processes (e.g. negative regulation of biological process, positive regulation of biological process, cellular protein modification process, developmental process, regulation of cellular metabolic process). Taking into consideration the importance of these SNP-affected biological processes in cell functioning, one may assume they may exert a substantial influence and be a source of inter-individual variability.

Nevertheless, the obtained results need further experimental validation to empirically shed more light on the functional implications of the SNP-caused alterations, especially since the exact functions of ssc-miR-9819-5p and ssc-miR-9791-1-3p have not been deciphered so far. The expression of these miRNAs has been detected in porcine milk exosomes^36^, and they are located in pig QTL regions associated with i.a. meat and carcass (e.g. meat pH, backfat thickness, longissimus thoracis muscle area, meat color), and production (e.g. feed intake and body weight), which suggests their engagement in economically important traits in pigs (PigQTLdb)^37,38^.

Most of the SNPs identified in this study are located beyond the seed region, which means that they do not exert direct influence on target gene selection via canonical binding. However, because they may influence the secondary structure of pre-miRNAs, they may have a substantial impact on the biogenesis of miRNAs^34^. To address this issue, we carried out the analysis of thermodynamical stability of the reference and SNP-altered pre-miRNAs with RNAfold software.

The analysis showed that 12 out of 65 identified SNPs do not cause any changes in the minimal free energy, meaning that they possibly do not change the stability of pre-miRNAs. 24 SNPs increased the MFE; thus, they have the potential to decrease the stability of the hairpins, while 29 SNPs decreased the MFE, which means they may stabilize the secondary structures. The average energy change of a secondary structure was 1.51 kcal/mol which is slightly lower from that established in humans (2.1 kcal/mol)^34^ and mice (2.17 kcal/mol)^35^. It may result from the fact that the aforementioned research teams calculated this mean energy change on the basis of all available wide-genome datasets from multiple studies.

It is worth to note that Gong and colleagues attempted to summarize the available research in this filed and stated that energy changes > 2.0 kcal/mol may significantly influence the mature miRNA production, but even energy changes below this threshold were observed to impact the miRNA biogenesis^34^. Therefore, identified in this study, energy changes may strongly affect pre-miRNA stability and miRNA production; however, only experimental validation will provide the ultimate evidence.

Moreover, our thermodynamical stability analysis revealed that 30 of the analyzed SNPs caused visible changes of the hairpin secondary structure, even without minimal free energy changes. It may be exemplified by ssc-miR-7141-5p, in which C>A SNP in the mature sequence created a small bulge and increased the MFE by 5.8 kcal/mol (Fig. 5). On the other hand, G>A SNP in the mature sequence of ssc-miR-9819-5p introduced only a 0.1 kcal/mol energy change, but it substantially affected the secondary structure (Fig. 6).

Further functional research is needed to confirm whether these predicted secondary structure changes have an impact on the miRNA biogenesis in the pig. However, they seem to have the potential to exert such an influence, especially in the light of the previous findings. Changes of the secondary structure introduced by an SNP were also reported by Harnprasopwat and colleagues, who investigated the human pri-miR-126^39^. Their analysis implicated that an SNP located in pri-miR-126 decreases the efficiency of further processing to the miRNA because secondary structure alterations affect Drosha cleavage. Similar findings were reported by Jazdzewski and colleagues, who showed that a common pri-miR-146a polymorphism influences pri-miR processing and protein binding to the pre-miR product^20^. Additionally, the research focused on the porcine miR-15b revealed that an SNP located in its pre-miR sequence exerts a profound influence, starting from pre-miR-15b processing, thermodynamical stability, secondary structure, through strand selection to the final level of expression^40^.

The conducted research not only tested the potential of targeted sequencing method for applications such as SNPs discovery in regions of interest, in this case microRNA genes, but also better characterized miRNA genes in terms of SNPs in the pig, and, at the same time, broadened the available repertoire of miRNA-localized SNPs. Thus, identified SNPs can be successfully used in future association studies. Moreover, the obtained results may facilitate pinpointing interesting miRNAs, which may become subjects of more extensive research, also those focused on associations with functional traits. It will reduce the cost of searching them de novo.

Large scale studies that is those investigating large research groups and large number of regions or even the whole genomes are very time- and cost-consuming. Therefore, studies on large groups are very often focused on only a few regions, so the number of identified SNPs is not very high; or concern species that are much more frequently and extensively studied, such as mice or humans. This is why livestock species, namely the pig, still stay behind when it comes to the number of miRNA localized SNPs; for example according to Zorc et al.^30^, the number of pre-miRNA polymorphic regions amounted to 1532 in human, 741 in cattle and as little as 89 in pigs (among which only 30 concerned mature miRNA sequences and seven embraced seed regions). Thus, broadening the knowledge in this field is essential and still a need, and our results with its 30 newly identified miRNA-SNPs make a substantial contribution, especially when taking into consideration the importance of miRNAs in cell functioning as well as their role as biomarkers. On the other hand, the identification of known SNPs constitutes additional confirmation and validation of the available data; especially since the used targeted method allowed obtaining very high read coverage, which very often cannot be achieved by whole genome sequencing at relatively low cost. Moreover, it also is crucial to broaden the SNP repertoire in as many different breeds as possible since SNPs’ occurrence differs between breeds, which also is beneficial for the state of knowledge and future association studies.

Altogether, the obtained results and analyses imply a great potential of SNPs to exert a multifaceted influence on the miRNA processing in the pig, which stays in agreement with the world-wide findings in different species. Furthermore, apart from broadening the available repertoire of porcine SNPs, they give a stimulus for further functional, extended research. To the best of our knowledge, we also report for the first time the use of the targeted sequencing approach for miRNA-genes focused studies. This technology appears as an interesting alternative for the whole genome sequencing, especially when regions of interest are limited.

## Material and methods

### Research material

The research material consisted of whole blood sampled of 23 pigs. The pigs belonging to the Polish synthetic line 990, which is a hybrid of several breeds (Large White, Belgium Landrace, Duroc, German Landrace, Walsh Landrace, and Hampshire), were maintained and slaughtered at the Pig Testing Station of the National Research Institute of Animal Production in Pawłowice under the same housing and feeding conditions. The approval of Animal Ethics Committee was not required because meat from slaughtered animals is standard intended for consumption.

### Targeted sequencing of microRNA genes

First, gDNA was extracted with the use of the QuickGene-Mini80 Nucleic Acid Isolation System (Kurabo) and QuickGene DNA Whole Blood Kit S (Kurabo), following the manufacturer protocols. The concentration and purity of the obtained isolates were assessed using the Qubit HS dsDNA Assay Kit (Thermo Fisher Scientific) and a Qubit 2.0 Fluorometer (Thermo Fisher Scientific), as well as a NanoDrop 2000 spectrophotometer (Thermo Fisher Scientific). Next, a custom targeted sequencing design was developed using SeqCap EZ Enrichment System provided by Roche Sequencing Solutions (Roche). The design and capture probe panel were prepared by Roche based on the genome coordinates of 381 pig pre-microRNA sequences deposited in miRBase (21.0)^41,42^. The total length of targeted regions was 109,204 bp, while the estimated coverage by the designed probes was 105,628 bp (96.7%). The single probe length was 100 bp, while the mean size of a captured region was 240 bp. Detailed data on the localization of the targeted regions are in the Supplementary File 1.

All targeted sequencing procedures were carried out following the SeqCap EZ HyperCap KAPA HyperPrep Workflow, including the double capture step and the multiplexing option. In brief, 700 ng of input gDNA for each sample was mechanically sheared with M220 Focused-ultrasonicator (Covaris) to obtain the average fragment size of 180–220 bp. The samples were ligated with adapters and subjected to dual size selection to get fragments between 250 and 450 bp. After PCR amplification and AMPureXp clean-up (Beckman Coulter), the libraries were pooled and hybridized for 16 h with the set of the custom-designed probes. The captured DNA was PCR amplified and cleaned up, and then subjected to the second hybridization, which was followed by the last PCR amplification and clean-up. Obtained libraries were checked for quantity and quality with the use of a NanoDrop 2000 spectrophotometer (Thermo Fisher Scientific), and a TapeStation 2200 system (Agilent). Finally, they were clustered on an Illumina Flowcell_v3 in a cBot cluster station and sequenced using the HiScan SQ (Illumina) system applying 94 cycles of single-end sequencing.

### Variant calling

Firstly, the quality of the raw reads was assessed with the FastQC software^43^. After quality control, the reads were filtered with Flexbar software^44^ by removing adapters, and reads of phread quality below 30, while the minimal read length was set to 50. Then, the mapping procedure was maintained with the use of BWA software^45^ followed by applying read groups to individual samples (Picard software^46^). After this step, the duplicate reads were marked (Picard software). The next step was the variant calling procedure with the use of Freebayes software^47^. After obtaining the raw vcf file, a series of filtering steps was applied with the use of VCFtools (minimal total depth above 5 (DP > 5), minimal genotype quality > 30)^48^. The obtained data were submitted to European Variation Archive (EVA) database (project PRJEB43246).

### In silico analysis of miRNA secondary structure and target selection

Pre-miRNA sequences identified in this study to have SNPs were analyzed with RNAfold software implemented in ViennaRNA package (version 2.4.13)^49^ to fold their secondary structures with and without the called SNPs, and calculate minimum free energy (MFE)^50^. The identification of target genes for miRNAs was performed using miRNAconsTarget tool (Miranda parameters) implemented in the sRNAtoolbox webserver and proving consensus target predictions^51,52^. The subsequent analysis of gene ontology enrichment was carried out with ShinyGO (v0.61) online tool applying the FDR correction for multiple testing^53^.

### Sanger sequencing

Primer pairs were designed using Primer3 software (v.4.1.0)^54^ to embrace 33 SNPs distributed in 20 genomic regions (Supplementary File 3). 100 ng of DNA was amplified using HotStarTaq polymerase (Qiagen). The amplicons were evaluated on a 3% agarose gel and cleaned up using ExoI and shrimp alkaline phosphatase (Thermo Fisher Scientific), according to the standard protocol. The purified products were sequenced using the BigDye Terminator v3.1 Cycle Sequencing Kit (Thermo Fisher Scientific) and primers used for the PCR amplification, following the manual. Unused reaction substrates were removed with the BigDye XTerminator Purification Kit (Thermo Fisher Scientific), according to the manufacturer protocol. The electrophoresis of the sequencing products was carried out on 3130xl Genetic Analyzer (Thermo Fisher Scientific). Base-calling was performed using Sequencing Analysis Software v5.2 (Thermo Fisher Scientific), while the obtained chromatograms were analyzed with FinchTV v1.4.0 software (Geospiza Inc.).

## Supplementary Information

Supplementary Legends.

Supplementary Information 1.

Supplementary Information 2.

Supplementary Information 3.
