# Supplementary material for: Application of the targeted sequencing approach reveals the single nucleotide polymorphism (SNP) repertoire in microRNA genes in the pig genome
Source: Sci Rep. 2021 May 10;11:9848. doi: 10.1038/s41598-021-89363-5 (PMC8110958; doi:10.1038/s41598-021-89363-5)
Supplement: Supplementary file 1 — Supplementary Legends. [file 41598_2021_89363_MOESM1_ESM.docx]

**Supplementary Materials: Supplementary File 1.** Detailed data on the localization of the designed capture probes in the pig genome. **Supplementary File 2.** Data on the genomic localization of the identified variants within miRNA sequences, along with the alleles and genotypes numbers and frequencies. The crossed out records represent sequences no longer present in the Sscrofa11.1 genome. **Supplementary File 3.** Table containing details on the validated regions, miRNAs and SNPs.
