# Supplementary material for: Application of the targeted sequencing approach reveals the single nucleotide polymorphism (SNP) repertoire in microRNA genes in the pig genome
Source: Sci Rep. 2021 May 10;11:9848. doi: 10.1038/s41598-021-89363-5 (PMC8110958; doi:10.1038/s41598-021-89363-5)
Supplement: Supplementary file 4 — Supplementary Information 3. [file 41598_2021_89363_MOESM4_ESM.docx]

**Application of the targeted sequencing approach reveals the Single Nucleotide Polymorphism (SNP) repertoire in microRNA genes in the pig genome**

Klaudia Pawlina-Tyszko^1*^, Ewelina Semik-Gurgul^1^, Artur Gurgul^1,2^, Maria Oczkowicz^1^, Tomasz Szmatoła^1,2^, Monika-Bugno-Poniewierska^3^

^1^Department of Animal Molecular Biology, National Research Institute of Animal Production, Krakowska 1, 32-083 Balice near Kraków, Poland.

^2^Center for Experimental and Innovative Medicine, the University of Agriculture in Kraków, Rędzina 1c, 30-248 Kraków, Poland.

^3^Department of Animal Reproduction, Anatomy and Genomics, the University of Agriculture in Kraków, al. Mickiewicza 24/28, 30-059 Kraków, Poland.

*Correspondence: [klaudia.pawlina@iz.edu.pl](mailto:klaudia.pawlina@iz.edu.pl)

| Region | SNPs localization | qPCR confirmed | miRNA | Primers 5’-3’ | Amplicon size |
| --- | --- | --- | --- | --- | --- |
| Region1  chr2: 6416400:6416850 | 6416616  6416639  6416695 | yes  yes  yes | - | F: TCAGCTCTGGTTCGGAAAGA  R: CAGATAACAGCAGCCCCACT | 399 bp |
| Region2 chr3:67137000:67137450 | 67137201  67137226 | yes  yes | - | F: AGTCTGGGTTTTGGCTAGGG  R: AGTCACCTCCTGTTTTCCCA | 389 bp |
| Region3 chr1:4108300:4108700 | 4108477  4108479 | yes  yes | ssc-miR-9831 | F: TCAGCTTTCAGTTCAAACAGCT  R: GGCAGTCTGTCTAAAGGGTAGA | 332 bp |
| Region4 chr1:301041900:301042400 | 301042126  301042136 | yes  yes | ssc-miR-9793 | F: CCGGGTCCCAAGTCAGGA  R: TGGGCATGGTCACTCATTCA | 368 bp |
| Region5 chr11:2111000:2111500 | 2111167  2111177  2111199 | no  no  no | ssc-miR-9814 | F: TCCATCCTCCTAGTTGAAGCC  R: GTGCTTCTCATGTTGCTGGT | 343 bp |
| Region6 chr12:57502300:57502700 | 57502421 | yes | ssc-miR-9853-1 | F: TCAACTGTGGGATATGGGGC  R: CTGAGGACATGAAGCAGCTC | 316 bp |
| Region7 chr3:22233150:22233650 | 22233442  22233454  22233461  22233462 | yes  yes  yes  yes | ssc-miR-9785 | F: TGGATTCACAAAACACTGCTCT  R: AGAGCTCAGAGTTCACCCTC | 305 bp |
| Region8 chr5:8348650:8349150 | 8348872  8348891  8348943 | yes  yes  yes | ssc-miR-9819 | F: GGCTTCTCCGGCTTTATGAG  R: GTTGGTGACGGGAGGAGG | 397 bp |
| Region9 chr7:123036400:123036900 | 123036545  123036556 | no  no | ssc-miR-10387 | F: AATGTCATCTTCTCCCCGCT  R: TGATGAACTCTACGGCAGCA | 328 bp |
| Region10 chr13:108388000:108388400 | 108388190 | yes | ssc-miR-15b | F: CTGCACACCCCGTAAAGTTT  R: TGCTATATCCCTGTCACGCT | 303 bp |
| Region11 chr6:52738800:52739200 | 52738915 | yes | ssc-miR-371 | F: GGTCCCTTGTGCATCTTCTC  R: TCACGGTCTGAGAAGTTGGA | 302 bp |
| Region12 chr4:93041100:93041500 | 93041219 | yes | ssc-miR-9849 | F:ACCCTAAGTGTGCTGATCAGT  R: AACACAGAGCCAGGGTAAATC | 301 bp |
| Region 13  chr2: 65581678:65582217 | 65581902 | yes | ssc-miR-23a | F: AGACCTCAAAGGATGGCAGC  R: AGTGGTAGGATAGGCAGGCA | 444 bp |
| Region 14  chr2: 12929686:12930146 | 12929925 | yes | ssc-miR-130a | F: ACGGAAGAAAAGGGAGGAAA  R: ACCAGGGTAGCTGATTGGTG | 274 bp |
| Region 15  chr9: 10799939:10800522 | 10800236 | yes | ssc-miR-326 | F: GTGTGAGTGCCCAGACCC  R: TTTCACAACCTCCCTGAGCC | 325 bp |
| Region 16  chr12: 38396445:38397028 | 38396767 | yes | ssc-miR-378-2 | F: CTGAGAGGGCAGAGCATAGG  R: CACAGGGAGCGTGGATATTT | 311 bp |
| Region 17  chr14: 123301603:123302206 | 123301846 | yes | ssc-miR-146b | F: TGAGAGAACTTTGGCCACCT  R: GTTGTCCCGGGTTACAGCTA | 309 bp |
| Region 18  chrX: 126199533:126199907 | 126199694 | yes | ssc-miR-19b-2 | F: TGTAGTGTGGGCACTTCCAG  R: CCGGGACAAGTGCAATACTT | 280 bp |
| Region 19  chrX: 126199578:126200098 | 126199852 | yes | ssc-miR-20b-1 | F: ACTGCCCTAAATGCCCCTTC  R: GCATGGATTTGCACAGCCAT | 386 bp |
| Region 20  chrX: 117607892:117608491 | 117608224 | yes | ssc-miR-2483 | F: TGGGTTCTCCATTCCCACCT  R: GTTTTTGCCACTGCTGAGGG | 521 bp |
